# Senescent T cells: Beneficial and detrimental roles

**DOI:** 10.1111/imr.13206

**Published:** 2023-04-25

**Authors:** Phatthamon Laphanuwat, Daniel Claudio Oliveira Gomes, Arne N. Akbar

**Affiliations:** ^1^ Division of Medicine University College London London UK; ^2^ Department of Pharmacology Faculty of Medicine, Khon Kaen University Khon Kaen Thailand; ^3^ Núcleo de Doenças Infecciosas Universidade Federal do Espírito Santo Vitoria Brazil; ^4^ Núcleo de Biotecnologia Universidade Federal do Espírito Santo Vitoria Brazil

**Keywords:** aging, senescence, T cell, TEMRA, terminally differentiated cell

## Abstract

As the thymus involutes during aging, the T‐cell pool has to be maintained by the periodic expansion of preexisting T cells during adulthood. A conundrum is that repeated episodes of activation and proliferation drive the differentiation of T cells toward replicative senescence, due to telomere erosion. This review discusses mechanisms that regulate the end‐stage differentiation (senescence) of T cells. Although these cells, within both CD4 and CD8 compartments, lose proliferative activity after antigen‐specific challenge, they acquire innate‐like immune function. While this may confer broad immune protection during aging, these senescent T cells may also cause immunopathology, especially in the context of excessive inflammation in tissue microenvironments.

## 1 INTRODUCTION

Aging is a dynamic process that progresses with concomitant deterioration of organ function that leads to loss of quality of life. Immunity also declines during aging. This is highlighted by the increased incidence of malignancy, loss of immunity to previously encountered pathogens, for example, varicella‐zoster virus (VZV) causing shingles,^1^, ^2^ decreased vaccine efficacy,^3^ and decreased ability to respond to new pathogens.^4^, ^5^ As the aging of cells in organs and of those within the immune system occur simultaneously, decreased immunity in an older individual may not result from a defect with a particular cell type instead, the problem may arise from altered interactions between aged immune and nonimmune cells in tissues.^6^ The term “cellular senescence” was established to describe aging of cell types and this process results in growth arrest that prevents the proliferation of cells harboring DNA damage and is therefore considered to be an anticancer mechanism.^7^ Senescent cells can be recognized by phenotypic and functional characteristics,^7^, ^8^ and these cells accumulate within both leukocytes (especially T cells) and nonimmune‐cell populations during aging.^9^, ^10^ Although senescent cells share many functional and phenotypic features, there are also cell‐specific differences.^11^, ^12^

One shared feature of different senescent cell types is their secretion of multiple cytokines, chemokines, enzymes, etc. (so‐called senescence‐associated secretory phenotype; SASP).^7^, ^8^, ^10^, ^13^ This may be a mechanism for altered communication between immune and nonimmune cells during aging.^8^ In this review, we focus on the development of senescent T cells and their phenotypic and functional features. We will also summarize recent information on how these cells may interact within an aged tissue environment. An emerging concept is that instead of being dysfunctional, senescent T cells begin to express the molecular machinery enabling them to behave more like natural killer (NK) cells.^6^ While this may confer them with broad nonantigen‐specific protection especially during aging, this may also result in immunopathology, where NK ligands are expressed by nonlymphoid cells within inflamed tissue microenvironments.

## 2 T CELL DIFFERENTIATION AND THE IDENTIFICATION OF SENESCENT T CELLS

**FIGURE 1:**
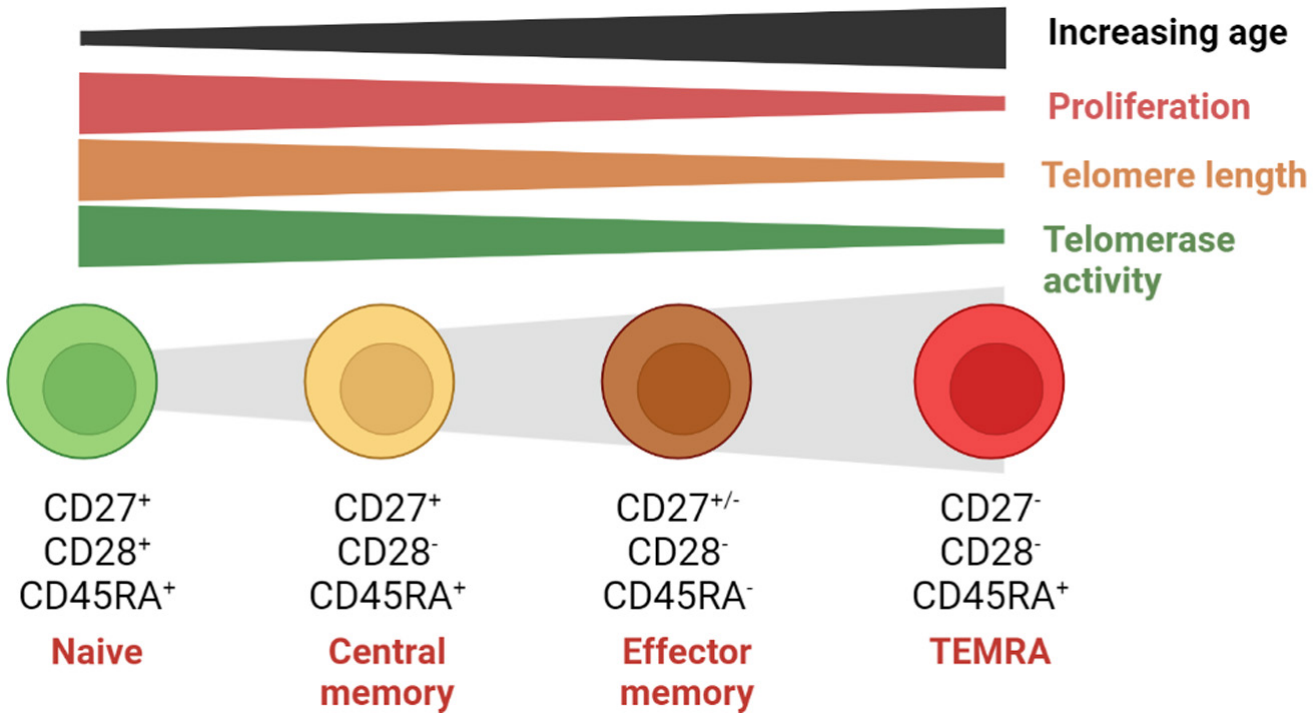
Schematic model for T cell differentiation during aging. Upon activation, naive T cells differentiate into several stages of memory and effector cells. The proliferative potential, telomere length, and telomerase activity are reduced upon differentiation across the lifetime. As age increase, the number of terminally differentiated cell TEMRA accumulates compared with other subsets. T cells lose the expression of costimulatory receptors CD27, CD28, and CD45RA as they differentiate from naïve to terminally differentiated cell. However, they regain expression of CD45RA when they reach an end‐stage and these (senescent) cells have limit replicative capacity but are highly cytotoxic and acquire NK‐related function.

A big advance in identifying T cells at early and end stages of differentiation was the combination of technology to identify telomeres together with cell surface in T‐cell subsets by multi‐parameter flow cytometry.^14^, ^15^, ^16^ This coordinate analysis enabled a pathway of differentiation of human T cells to be defined (Figure 1). Hence, while naïve T cells in both CD4^+^ and CD8^+^ compartments express the co‐stimulatory receptors CD28 and CD27 and have long telomeres, end‐stage T cells lose expression of these molecules and shorten their telomeres considerably.^17^, ^18^ Furthermore, some effector memory (EM) T cells that are CD27^−^CD28^−^ re‐express cell surface CD45RA and are known as the EMRA subset.^19^, ^20^ Once the definition of the differentiation states of T cells was made, it enabled early and end‐stage cells to be isolated and their functional characteristics to be assessed. Thus naïve, central memory and effector cells have different functions and homing potential and tissue localization.^21^ In human peripheral blood, CD27^−^CD28^−^ T cells within both CD4^+^ and CD8^+^ compartments express multiple cell surface markers of T cells senescence including KLRG1 and CD57 and also intracellular molecules associated with cell cycle arrest and senescence (p16 and p21)^20^, ^22^, ^23^ (Figure 1). This was confirmed by single‐cell RNAseq analyses of human CD8^+^ T cells.^24^ We henceforth will refer to the CD27^−^CD28^−^ T‐cell population as senescent T cells. Of note, murine CD4^+^ and CD8^+^ T‐cell populations do not lose the expression of CD28 as they undergo repeated rounds of proliferation indicating that there are key differences in differentiation‐related phenotypic changes between humans and mice.^25^ It is recognized widely that while the proportion of naïve T cells decreases during aging, the proportion of senescent cells increases, due in part to activation by persistent viruses such as cytomegalovirus (CMV) in vivo.^26^, ^27^, ^28^

## 3 THE PROLIFERATIVE POTENTIAL OF T CELLS

The loss of thymic activity in early life results in a considerable reduction in the production of new naïve T cells.^29^ Thus, in maturity, the T cell repertoire is fairly rigid as there is decreased production of T cells with novel specificities.^30^ This may explain why older subjects become very susceptible to pathogens that have not been encountered previously. However, this also raises a conceptual problem, that is, how are T cell numbers maintained throughout life without any new input of thymically derived naïve cells? The repeated rechallenge by antigen can maintain the high precursor frequency of antigen‐specific T cells in vivo.^31^ In addition, the homeostatic proliferation driven by cytokines such as IL‐7 (for CD4^+^ T cells) and IL‐15 (for CD8^+^ T cells) can expand T cell populations irrespective of their antigen specificity.^32^, ^33^, ^34^, ^35^ However, the continuous proliferation of T cells through life may lead to loss of proliferative potential called replicative senescence that is due to telomere erosion^36^ raising the question of whether this could lead to a loss of immune memory to frequently encountered antigens.^37^, ^38^ However, the loss of telomeric DNA can be mitigated if cells are able to upregulate the enzyme telomerase, which can replenish telomeres.^39^ Nevertheless, T cells lose the capacity to upregulate this enzyme after repeated rounds of proliferation.^25^ This raises the question of whether the loss of telomerase activity and the reduced capacity to divide after activation is a fixed feature of end‐stage differentiation (senescence) of T cells or if this process is actively controlled by cell‐signaling pathways and may be reversed, with implications for enhancing immunity during aging.

A novel mechanism for telomere maintenance that is independent of telomerase was described recently when antigen‐presenting cells donate telomeres to T cells, enabling them to maintain their replicative capacity and responsiveness to antigen stimulation.^40^ This telomere elongation mechanism occurred in humans and mice and involved the transfer of the telomeres together with DNA recombinant factor Rad51 via extracellular vesicles from antigen‐presenting cells, enabling the donated telomeres to recombine with the ends of T‐cell chromosomes. This resulted in the elongation of recipient T cell telomeres by ~3000 base pairs. Interestingly, this transfer mainly occurred in naïve and central memory T cells and to a considerably reduced extent in effector T‐cell populations.^40^ This indicates the existence of different cell fates after activation, whereby telomerase activation and telomere transfer may preserve the proliferative potential of some cells, whereas others are destined to reach an end stage (senescence) and lose their ability to proliferate.^20^, ^37^

End‐stage (senescent) T cells that have shorter telomeres in the steady‐state accumulate in vivo in healthy individuals during aging^18^ and in patients with genetic defects that induce an exuberant T‐cell proliferative response after stimulation, for example, X‐linked lymphoproliferative syndrome.^41^ In addition, many diseases are associated with the accumulation of end‐stage T cells, and it is not clear if these cells are causative or arise as a consequence of the disease state (Table 1). T cells that are specific for certain persistent infections such as that induced by cytomegalovirus, exhibit disproportionate telomere erosion compared with T cells that are specific for other persistent viruses (Epstein–Barr virus, varicella‐zoster virus, herpes simplex virus).^42^ Thus, after a single episode of immune stimulation, a dichotomy of T cell fates may be induced with a proportion of cells retain replicative potential, whereas others differentiate toward senescence and lose their replicative capacity but are able to persist in vivo. Both types of T cells may be important for immune protection, and they coexist in healthy individuals and patients with various diseases.

**TABLE 1:** List of putative beneficial roles of senescent T cell and their senescence‐associated marker expression.

Roles	Identification of senescent cells within T cell compartment	Mechanism and impact on protective immunity	Species	References
Tumor immunity (disease)				
Advanced melanoma	CD4^+^ CTLs (CD4^+^CRTAM^+^CX3CR1^+^)	Cytotoxic activity mediated by CD4^+^ T cells against human melanoma cell line	Human	[86, 169, 170]
Bladder cancer	CD4^+^CCR7^−^CD45RA^+^	Cytotoxic activity mediated by EMRA CD4^+^ T cells	Human	
	CD8^+^CD39^+^	CD4^+^ T cells mediate autologous tumors killing in an MHC class II‐dependent fashion	Human	
Antiviral immunity				
Dengue virus	CD4^+^CX3CR1^+^CCR7^−^CD45RA^+^	Direct ex vivo DENV‐specific cytolytic activity	Human	[92, 94]
Influenza virus	CD4^+^ CTLs (CD4^+^GrzB^+^)	CD4^+^ cells acquire perforin‐mediated cytotoxicity in the lung that may enhance recovery from lethal infection	Mouse	
Antiparasitic immunity				
Malaria	CD4^+^ CTLs	Transfer of CD4^+^ Cytolytic T cell conferred Protection	Mouse	[93, 171]
	CD4^+^CD38^+^	Reduced plasmodium parasite burden associates with cytolytic CD4 cells	Human	

In a recent study, T cell proliferation was determined in mice that were primed and boosted twice in vivo, then these cells were isolated from spleens and transferred to new mice; this sequential process of priming and boosting was repeated 51 times over 10 years.^43^ Despite the tremendous expansion that the antigen‐specific T cells occurred as a result of this repeated challenge, they retained their capacity to respond to the original antigen.^43^ Therefore, previous models of the constrained replicative life span of T cells after repeated challenge considerably underestimated their true capacity for expansion.^44^, ^45^, ^46^ T cells required periods of rest (~30 days) between restimulations to maintain this replicative capacity, and shorter periods of time between rechallenge (7 days) led to the loss of replicative potential. In these experiments, the antigen‐specific T cells maintained their telomere length despite the substantial expansion in vivo.^43^

Mice have 10‐fold longer chromosomal telomeres than humans.^47^ This indicates that the impact of telomere erosion on the expansion of T cells over a mouse life span will be less than that observed in humans.^47^ The maintenance of long‐term proliferative capacity of T cells^43^ may therefore be due to long initial telomeres, the novel telomere transfer mechanism in vivo^40^ and possibly continued activation of telomerase after activation (not assessed in the study of Soerens et al.^43^) or a combination of all three. While the T‐cell transfer experiments showed the impressive ability of these cells to expand beyond the life span of an individual mouse, the generation of end‐stage like T cells after each round of stimulation was not assessed.^43^ As the T cells were repeatedly transferred to young mice, the impact of an aged tissue environment on T cell fate after activation was not determined. Therefore, the maintenance of replicative capacity of T cells in these experiments does not reflect T cell expansion in vivo during aging. In humans and mice, end‐stage effector cells are generated during an immune response, and the majority of these cells are cleared upon immune resolution.^48^ Nevertheless, T cells with phenotypic and end‐stage characteristics (discussed below) accumulate in both humans and mice during aging.^9^, ^49^ A precise molecular identification of end‐stage human T cells and their functional attributes has not been made and the consequences of the accumulation of these cells during healthy aging and in a range of diseases is not known.

## 4 HOW IS THE FUNCTION OF SENESCENT T CELLS REGULATED?

Senescent T cells within both CD4 and CD8 compartments exhibit low proliferative activity and telomerase induction after T‐cell receptor activation.^18^, ^22^ However, these cells are highly secretory, expressing high constitutive levels of inflammatory cytokines including IFN‐ɣ and TNF‐𝝰 and also the cytotoxic proteins perforin and granzyme.^34^, ^50^, ^51^ This is reminiscent of the senescence‐associated secretory phenotype (SASP) that is exhibited by nonsenescent lymphoid cells although with shared but also distinct secreted mediators.^7^ In healthy humans, these senescent cells coexist with naïve and central memory T‐cell populations that retain their proliferative potential.^38^, ^43^

It is unclear if the reduced proliferative capacity and telomerase activity of senescent cells were a permanent or a reversible functional feature of human T cells. The proliferative activity of senescent CD8^+^ T cells could be enhanced after T‐cell stimulation either by blocking the PD‐1 exhaustion‐related inhibitory receptor signaling pathway, using antibodies against the ligand (PDL‐1/PDL‐2) that is found on antigen‐presenting cells or by blocking p38 MAP kinase signaling using a small‐molecule inhibitor.^22^, ^52^ However, only p38 MAP kinase but not PD‐1 inhibition could reconstitute telomerase activity.^52^ This indicated that key functional changes in senescent T cells were mediated by active cell‐signaling processes rather than a passive loss of function and this loss of activity was reversible. Senescent CD4^+^ and CD8^+^ T cells showed constitutive expression of p38 MAP kinase and this was induced by a novel mechanism involving AMP kinase and TAB1.^54^ Furthermore, this non‐canonical pathway was activated by either DNA damage‐induced senescence or glucose deprivation. This showed a convergence of senescence signaling and nutrient sensing pathways in inducing the inhibition of the function of senescent T cells.^54^

## 5 THE IDENTIFICATION OF A NOVEL MULTI‐PROTEIN INHIBITORY COMPLEX IN SENESCENT CD4 ^+^ T CELLS

In addition to p38, there are two other subgroups of MAPKs namely Erk and Jnk.^55^, ^56^ Collectively, these signal‐transducing enzymes are involved in a wide range of mammalian physiology, including senescence, aging, and metabolism.^57^ It was considered that each MAPK was regulated independently in different cell types.^58^, ^59^, ^60^ Since p38 MAPkinase could regulate the function of senescent T cells, it raised the question of whether Erk and Jnk also had a role in regulating the function of this population. It was therefore of considerable interest that the p38, Erk, and Jnk MAPK subgroups were colocalized within an individual inhibitory signaling complex in senescent CD4^+^ T cell‐containing AMPK, that also contained the stress proteins known as sestrins.^56^ Sestrins are the mammalian products of the *Sesn1*, *Sesn2*, and *Sesn3* genes.^61^ Sestrin expression is induced upstream of AMPK activation in senescent CD4^+^ T cells.^56^ This novel sestrin‐activated MAPkinase activation complex (called an sMAC) was identical in senescent humans and in old (20 months) murine CD4^+^ T cells.^56^ Furthermore, this complex operated independently of mTOR activity.^56^ The expression of sestrins was increased in both older humans and mice and the blocking of this complex enhanced antigen‐specific proliferation of senescent CD4^+^ T cells in humans in vitro and in CD4^+^ T cells mice in vivo.^54^, ^56^ Although this sestrin complex coordinates the simultaneous activation of all 3 MAP kinases in senescent T cells, each MAPkinase, once activated, controlled a distinct functional response. However, the disruption of global sMAC signaling restored antigen‐specific proliferation and cytokine production in CD4^+^ T cells from old humans and enhanced responsiveness to influenza vaccination in old mice.^56^ This raises the question of whether the sestrins also regulated the function of senescent CD8^+^ T cells.

## 6 THE REGULATION OF SENESCENT CD8 ^+^ T CELL FUNCTION BY SESTRINS

Single‐cell RNAseq analyses of human CD8^+^ T cells confirm that loss of costimulatory molecules (CD27 and CD28) and acquisition of KLRG1 and CD57 receptors are found in the same cells that express senescence characteristics.^24^ Furthermore, the senescent CD8 population also expresses high levels of sestrins compared with the nonsenescent population.^24^ Thus, senescent T cells in both CD4 and CD8 compartments can be identified by the same phenotypic characteristics. A significant increase in senescent‐like CD8^+^ compared with CD4^+^ T cells has consistently been observed in healthy old subjects.^53^, ^62^, ^63^ Single‐cell analyses also showed that senescent CD8^+^ T cells express a wide range of activating and inhibitory NK receptors and their adaptor molecules,^24^ confirming previous reports.^64^ Human senescent CD8^+^ T cells can kill NK target cells in vitro and whole CD8^+^ T cells from old mice, that also express NK receptors can kill NK target cells in vivo.^24^

Natural killer‐like senescent CD8^+^ T cells also lose TCR signaling‐related molecules and reduced TCR signaling‐induced proliferative activity. A key observation was that the transition from TCR to NKR‐related function is reversible by blocking the sestrins.^24^ Therefore, senescent CD8^+^ T cells do not lose functionality, instead, they have altered functional capabilities that may enable them to provide broad rather than antigen‐specific immune protection, for example, against tumors or infected cells. In this context, the accumulation of these cells in older individuals, perhaps resulting from a persistent viral infectious challenge, may be beneficial for the host although this point has not been directly proven (Table 1).

## 7 INTERACTION BETWEEN SENESCENT STROMAL VERSUS IMMUNE CELLS

Senescent nonlymphoid cells in tissues can be recognized and eliminated by the immune system.^65^, ^66^, ^67^ Different immune cell types including macrophages, neutrophils, natural killer (NK) cells, and CD4^+^ T cells may be involved in this surveillance of senescent cells, depending on the pathophysiological context.^68^, ^69^, ^70^, ^71^, ^72^ Senescent stromal cells express stimulatory ligands like MICA/B that bind to NKG2D which induce their killing by NK cells.^69^, ^73^ Moreover, through the secretion of SASP‐related products such as chemokines and cytokines, senescent cells can recruit immune cells into tissues to facilitate senescent tissue cell clearance.^70^, ^74^ Through this senescence–associated secretory process, a low‐level chronic inflammatory state may develop that underlies many age‐related diseases.^75^, ^76^ Despite the evidence for senescent cell clearance by the immune system, it is not known why senescent cells accumulate during aging and persist at sites of age‐related pathologies.^77^ An overall decline in innate and adaptive immune responses during aging may contribute to the incomplete elimination of senescent cells.^9^, ^78^ Alternatively, changes in major histocompatibility complex (MHC) expression can lead to evasion by senescent cells from recognition by the immune system as previously described in cancer and in virally infected cells in vivo.^79^, ^80^, ^81^

Aging is associated with the accumulation of senescent structural cells within tissues that secrete a wide range of pro‐inflammatory mediators. Therefore, immune responses that take place in the tissue environments of older individuals will involve the interaction between old (senescent) leukocytes and old tissue cells that will be considerably different from immune responses in tissues of younger individuals. Senescent fibroblasts express MHC class I chain‐related proteins A and B (MICA/B) which is a ligand for NKG2D, an interaction that activates NK cytotoxic function.^69^, ^73^ These senescent stromal cells also express a nonclassical MHC class Ib molecule HLA‐E that binds to the inhibitory NK receptor NKG2A that inhibits NK killing.^6^ It has been shown that inhibitory NK receptor signaling overrides the function of activating NK receptors^82^, ^83^ and activating and inhibitory NK ligands are expressed simultaneously by senescent fibroblasts both in vitro and in vivo.^6^ These fibroblasts could be killed by both NK cells and CD8^+^ T cells via NKG2D–MICA/B interactions, however, the inhibition of interaction between the inhibitory NK‐receptor NKG2A with HLA‐E could enhance this killing activity.^6^ There is increased expression of the inhibitory NK‐ligand HLA‐E in the skin during aging and this has been suggested to be one reason that senescent cells accumulate in the tissues of older individuals.^6^

## 8 IS THE ACCUMULATION OF SENESCENT T CELLS DURING AGING OF BENEFIT TO THE HOST?

**FIGURE 2:**
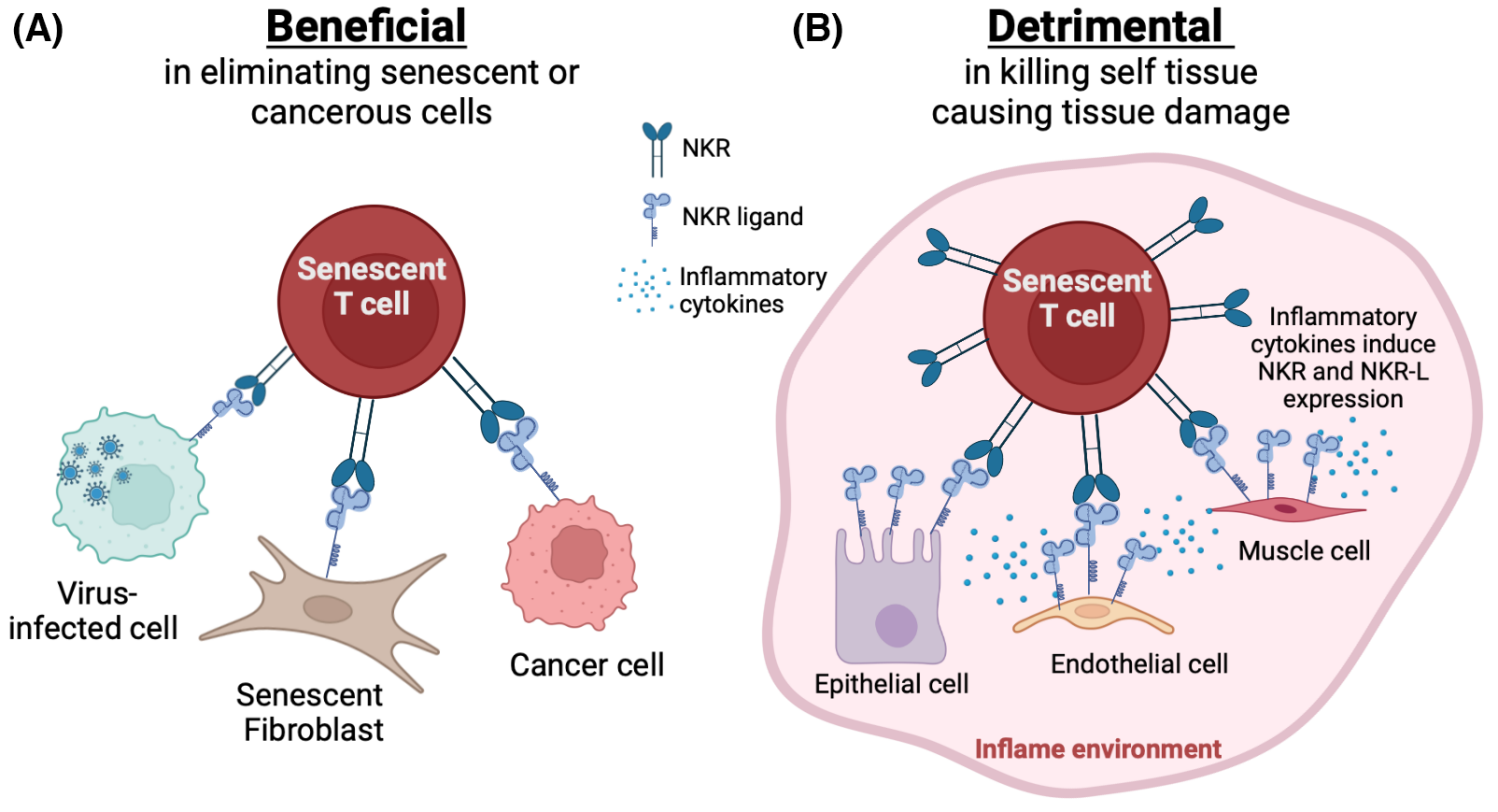
Beneficial and detrimental roles of senescent T cells. (A) Senescent CD8^+^ T cells express NK cell receptors (NKR) such as NKG2C and NKG2D that recognize their ligands (NKR‐L) such as MICA/B that is expressed by on the senescent stromal cells, tumor cells, and and virally infected cells, that contributes to their clearance. (B) Overwhelming inflammatory cytokine production drives the ability of senescent T cells to recognize healthy tissue cells (e.g., epithelial, endothelial, muscle and immune cells) that are induced to express stimulatory NK ligands. This interaction may promote the aberrant killing in a nonspecific fashion and contribute to off‐target tissue pathology.

Both NK cells and CD8^+^ T cells have been shown to kill autologous senescent fibroblasts in vitro.^6^ As the accumulation of senescent tissue cells has a detrimental impact on organ function and removal of senescent cells in vivo reverses age‐associated pathology, the accumulation of senescent T cells during aging may be beneficial as they can eliminate senescent nonlymphoid tissue cells. Therefore, in addition to the broad nonantigen‐specific protection against malignancy and infection that senescent CD8^+^ T cells may provide, they may also reduce organ‐specific dysfunction in older individuals by clearing senescent cells from tissues^84^, ^85^ (Figure 2).

Senescent‐like CD8^+^ T cells can kill tumor targets in vivo.^24^ A literature search has identified a wide range of diseases where senescent T cells accumulate (Table 1). It has been suggested that senescent CD8^+^ T cells play an essential role in the immunosurveillance of malignant cells.^24^ Cytotoxic CD8^+^ T cells are the majority of tumor‐infiltrating lymphocytes (TILs)^84^ and these cells express a CCR7^−^CD45RA^+^ (or TEMRA) phenotype in nonsmall‐cell lung cancer (Table 1). Interestingly, TILs with a TEMRA phenotype have transcriptional differences from their peripheral blood counterparts. Similarly, senescent‐like CD4^+^ T cells with cytotoxic activity (CD4^+^ CTL) have been implicated in protection against malignancy.^86^ These cells can also secrete IFN‐γ and TNF‐α, granzyme B^87^, ^88^ that may support the tumor clearance. Senescent‐like CD4^+^ cytotoxic T cells in cancer patients downregulate CD27 and CD28 and upregulate the expression of CD57 and NK cell receptors such as NKG2C, NKG2D, and KIR.^89^, ^90^ This indicates that they may be very similar to NKR‐expressing senescent CD8^+^ T cells in humans but are found in much lower numbers.^6^ The expression of NK receptors would allow senescent‐like CD4^+^ cytotoxic T cells to kill in an antigen nonspecific manner, however, they may also be triggered in an antigen‐specific manner by activation via TCR‐MHC class II.^91^

Senescent‐like CD4^+^ CTL also expands following infections, suggesting their involvement in both antiviral and antiparasitic immunity^92^, ^93^, ^94^ (Table 1). The expansion of this subpopulation correlates with the viral load control observed in patients with HIV^95^, ^96^ and influenza virus infection.^97^ A direct protective role for these cells was shown in murine models, where CD4^+^‐CTL populations were associated with protection against lethal influenza virus challenge.^98^ In addition, they contribute to B‐cell maturation and antibody production and perforin‐mediated cytolytic activity, which was associated with antiviral protective immunity.^98^ The expansion of CMV‐specific CD4 T cells with an effector memory phenotype and increased inflammatory capacity as well as GrzB have been associated with better clinical outcome for patients.^99^ An interesting observation is that following the reduction of viral load in primary CMV infection, a population of CD28^−^CD27^−^ CD4 T cells expressing perforin and GrzB emerged in the circulation of infected individuals concomitantly with senescent CD28^−^CD27^−^ CD8^+^ T cells.^100^ In addition, both CD4^+^ and CD8^+^ T cells that are CD27^−^CD28^−^ subsets are associated with viral latency. Thus, senescent T cells with broad NK‐like functions may be beneficial for broad antiviral and antitumor protection and also for the clearance of senescent nonlymphoid cells in tissues.

## 9 A PUTATIVE ROLE FOR SENESCENT T CELLS IN INDUCING PATHOLOGY

There is a wide range of diseases where senescent T cells accumulate (Table 2). These cells may directly or indirectly contribute to the disease process.

**TABLE 2:** List of detrimental roles of senescent T cell and their senescence‐associated marker expression.

Disease	Identification of senescent cells within T‐cell compartment	Mechanisms and impact in pathology	Species	References
Autoimmune diseases				
Amyotrophic lateral sclerosis (ALS)	CD8^+^ EMRA (CD45RA^+^CD27^−^)	Increased frequencies of antigen‐specific CD8^+^ T cells mirror the progression of motor neuron disease and correlate with anti‐glioma immunity	Mouse	[172]
Grave's orbitopathy	Diverse (Increased KLRG1^+^, GrzB^+^)	Possible direct—Cytotoxic CD4^+^ T cells with increased inflammatory capacity were involved in orbitopathy activity	Human	[173]
Lupus nephritis	CD4^+^ CD28^−^	Renal injury mediated by cytotoxic activity	Human	[35]
	CD8^+^ CD28^−^	Significant positive correlation between increasing absolute frequencies of CD3^+^CD8^+^CD28^−^ cells and disease activity	Human	[174]
	CD4^+^CD28^−^	CD4^+^ cytotoxic cells with migratory capacity and increased cytotoxic potential	Human	[136, 175]
Multiple sclerosis	CD4^+^ CTLs and CD8^+^ EMRA	Abnormally increased frequencies of CD4^+^ T cells with activated and cytotoxic effector profiles may be implicated in progressive disease.	Human	[135]
Psoriatic arthritis	CD8^+^ EMRA (CD45RA^+^CCR7^−^)	T cells showed increased capacity to migrate and accumulate into synovial fluid obtained from PA patients	Human	[176]
Rheumatoid arthritis	CD4^+^CD28^−^	Circulating CD4^+^CD28^null^ lymphocytes are increased in RA patients	Human	[177]
	CD4^+^CD28^−^	Highly differentiated CD4^+^‐CTL expanded and were associated with disease severity	Human	[167]
Sjogren's syndrome	CD4^+^‐ and CD8^+^‐CTLs	Cytotoxic cells may be of pathogenic relevance in directing tissue damage in SS	Human	[178]
Systemic sclerosis	CD4^+^ CTLs CD57hi	Cytotoxic T cells may induce the apoptotic death of endothelial and other cells in systemic sclerosis	Human	[179]
Non‐infectious diseases				
Acute coronary syndrome	CD4^+^CD28^−^	Markedly augmented CD28^null^ T‐cell cytotoxic function and interferon‐γ production	Human	[180]
Acute heart failure	CD4^+^CD57^+^	Senescent T cells showed more inflammatory features and polyfunctionality and were associated with clinical outcome in patients with acute HF	Human	[147]
Kidney transplant	CD8^+^ TEMRA	Inflammatory and migratory role of TEMRA CD8^+^ T cells in humoral transplant rejection. Kidney transplant recipients may benefit from therapeutics targeting these cells.	Human	[181]
	CD8^+^ EMRA	TEMRA CD8^+^ T cells play a pivotal role in humoral and cellular rejection and reveal the potential value of memory CD8^+^ T cell monitoring for predicting risk of kidney transplant failure.	Human	[183]
	CD8 EMRA (CD45RA^+^CD28^−^)	TEMRA CD8^+^ from KT respond vigorously to IL‐15 stimulation and foster the endothelium inflammation by the upregulation of CX3CL1 on human umbilical vein endothelial cells (HUVECs) through the secretion of IFN‐γ and TNF‐α.	Human	[183]
Type 2 diabetes	CD4^+^ and CD8^+^ EMRA (CD45RA^+^CCR7^−^)	Increased number of senescent T cells in both the CD4^+^ and CD8^+^ T‐cell compartments. These senescent T cells show impaired migratory capacity despite having an upregulated chemokine receptor expression.	Human	[134]
Infectious diseases				
Acute hepatitis A	CD8^+^ NKG2D^+^, NKp30^+^	CD8^+^ T cells specific to HAV‐unrelated viruses are activated and proliferate during AHA. Hepatocytes from HAV‐infected liver overexpressed NKG2D ligands. Liver injury during AHA is associated with innate‐like cytotoxic function of bystander‐activated CD8^+^ T cells in an NKG2D‐dependent manner		[149]
CMV	CD8^+^ EMRA (CD45RA^+^CD28 low)	CD8^+^ T cells specific to HAV‐unrelated viruses are activated and proliferate during AHA. Hepatocytes from HAV‐infected liver overexpressed NKG2D ligands, making them potential cytolytic targets of bystander‐activated HAV‐unrelated virus‐specific CD8^+^ T cells. Liver injury during AHA is associated with innate‐like cytotoxic function of bystander‐activated CD8^+^ T cells	Human	[185]
	CD4 CD28^−^, CD27^−^, GrzB^+^, perforin^+^	CD4^+^CD28^−^ T cells emerge following primary CMV infection, indicating that infection triggers the formation of this subset. CD4^+^CD28^−^ cells had an Ag‐primed phenotype and expressed the cytolytic molecules granzyme B and perforin.	Human	[185]
	CD4 CD28^−^, CX3CR1^+^, NKG2D^+^, perforin^+^	CD4^+^CD28^null^ cells were found predominantly in CMV‐seropositive patients and expanded in the post‐transplantation period. This subset was predominantly effector memory phenotype and expressed markers of endothelial homing (CX3CR1) and cytotoxicity (NKG2D and perforin)	Human	[186]
Covid	CD8^+^ EMRA (CD45RA^+^ CD27^−^)	SARS‐CoV‐2 infection was associated with EMRA CD8^+^ T cell activation in a subset of patients	Human	[151, 152]
	CD8 KIR^+^ NKG2A^−^ CD45RA^+^	KIR^+^RA^+^ T cells were a major T‐cell subset becoming activated in older adults suffering from an acute respiratory viral infection	Human	
Cutaneous leishmaniasis	CD4^+^ and CD8^+^ EMRA (CD45RA^+^CD27^−^)	Senescent T cells accumulate in blood of patients with CL and correlate with lesion size	Human	[118, 119]
	CD4^+^ and CD8^+^ EMRA (CD45RA^+^CD27^−^)	Lesional accumulation of senescent CD8 T cells correlate with lesion size	Human	
Human papillomavirus	CD4 CD28^−^ NKG2D^+^	Increased frequency of CD4^+^NKG2D^+^ T cells in patients with cervical intraepithelial neoplasia grade‐1	Human	[143]
Influenza B	CD8 KIR^+^ NKG2A^−^ CD45RA^+^	KIR^+^RA^+^ T cells were a major T‐cell subset becoming activated in older adults suffering from an acute respiratory viral infection	Human	[152]
Dengue	CD4 Perforin^+^	CD4^+^ CTL clones were demonstrated to lyse cognate antigen‐presenting target cells by a perforin production, while bystander lysis occurred through Fas/Fas ligand interactions. CD4^+^ T‐cell clones also produced gamma interferon, tumor necrosis factor alpha (TNF‐α) and TNF‐β	Human	[187]
Seasonal coronavirus	CD8 KIR^+^ NKG2A^−^ CD45RA^+^	KIR^+^RA^+^ T cells were a major T‐cell subset becoming activated in older adults suffering from an acute respiratory viral infection	Human	[152]

### 9.1 The impact of CMV infection on driving T‐cell senescence

Memory T cells that are specific for persistent antigens that are encountered frequently are driven to differentiate continuously toward an end stage. Despite this, certain viruses have a disproportionate impact on driving T cells toward an end stage or senescence. One such virus is cytomegalovirus (CMV) which induces the preferential expansion of specific CD4^+^ and CD8^+^ T bearing CD27^−^CD28^−^ and/or TEMRA phenotype even compared with T cells that are specific for other persistent viruses such As Epstein–Barr Virus (EBV) and varicella‐zoster virus (VZV) antigens like in the same individuals.^42^, ^101^ While there are some studies that suggest that persistent CMV infection is associated with decreased 2‐ and 4‐year survival of subjects over 80 years of age,^102^ did not show an association with CMV and all‐cause mortality.^103^ Nevertheless, persistent CMV infection may impair immunity to other viruses such as EBV^104^ and may also contribute to “inflammaging” and greater frailty during aging.^105^ Therefore, while persistent CMV infection may not affect the life span of older subjects, it may have either a direct or indirect impact on their health. In contrast to observation in older subjects, CMV infection may actually have a beneficial effect on boosting influenza vaccination responses in younger individuals.^106^ The question is whether the expanded CMV‐specific effector CD8^+^ T cells that are found in older subjects^28^, ^101^ have an impact on health span. Since aging and increased low‐grade systemic inflammation are linked,^76^, ^107^ the expanded population of end‐stage (senescent) CMV‐specific CD8^+^ T cells as well as CD4^+^ CTL that may express NK receptors can target healthy tissues expressing NK ligands as a result of inflammageing; this process may trigger chronic nonspecific tissue damage.

### 9.2 The accumulation of senescent CD8^+^ T cells in patients with cutaneous leishmaniasis

Cutaneous leishmaniasis (CL) is considered a severely neglected parasite infection, causing immune‐mediated skin pathology characterized by the development of destructive cutaneous lesions.^108^ The skin lesions emerge at the parasite inoculation site usually after 2–4 weeks and may become chronic. Previous studies have hypothesized that the elevated parasite load and antigenic abundance fuel the inflammatory response, leading to ulcer development^109^ Thus, the development of the classical ulcer occurs concomitantly with the production of both gamma interferon (IFN‐γ) and tumor necrosis factor‐alpha (TNF‐α), and both positively correlate with lesions size and severity of the disease observed in the patients.^110^, ^111^

Substantial evidence has also linked the role of cytotoxic cells in mediating the skin pathology of human infection caused by *Leishmania*.^112^ CD8^+^ T cell activity increases as the infection progresses.^113^, ^114^, ^115^ In addition, the severity of the disease and the number of CD8^+^ T cells present in the lesions are linked significantly,^113^, ^116^ and this is independent of the parasite burden within the lesions.^117^ This raises the question about the cause of the lesions in the skin, suggesting the possibility that chronic inflammation and nonspecific cytotoxic responses may lead to nonspecific tissue destruction.

Individuals infected with *L. braziliensis* have elevated numbers of circulating CD4^+^ and CD8^+^ T cells that have characteristics of senescence, including short telomeres, decreased expression of the catalytic component of the enzyme telomerase (hTERT), reduced proliferative activity, and increased expression of the nuclear DNA damage response protein H2AX.^118^ Furthermore, T cells in the infected patients secrete SASP‐like cytokines in response to *L. braziliensis* antigen (LbAg) or anti‐CD3 stimulation compared with controls in vitro. Although senescent CD8^+^ T cells accumulate in patients,^118^ the robust proliferative response to LbAg stimulation indicates that not all the *L. braziliensis*‐specific T cells are senescent^111^ supports the contention that senescent and nonsenescent T cells coexist in vivo.

The positive association between the frequency of circulating senescent T cells within both CD4^+^ and CD8^+^ compartments and cutaneous lesion size suggests that these cells may be recruited into the lesions and contribute to skin ulceration.^118^, ^119^ This is further supported by the fact of elderly patients, who have more circulating senescent T cells have larger cutaneous lesions and longer duration of illness than young patients. Moreover, they are more likely to develop mucocutaneous leishmaniasis, the most inflammatory and severe clinical form of the disease.^120^, ^121^, ^122^

Senescent T cells from CL patients but not healthy controls significantly upregulate the skin‐homing receptor CLA in response to *L. braziliensis* antigens recall that facilitates their homing into the skin.^118^ Transcriptomic analyses demonstrate a strong co‐induction of senescence and pro‐inflammatory gene signatures in cutaneous leishmaniasis (CL) lesions,^123^ which has been linked to CD8^+^ effector memory, TEMRAs, and senescent NK cells,^124^ thus supporting a role for senescent cells in the immunopathology of LC. CD4^+^ T cells with senescence characteristics also accumulate in lesions and express NKR such NKG2C and NKG2D (L. P. Covre, R. G. de Moura, C. H. Fantecelle, P. de Oliveira Lopes, A. Falqueto, A. N. Akbar, & D. C. O. Gomes, unpublished). Interestingly, this population showed a conspicuous cytotoxic capacity and ability to mediate the lysis of target cells in an antigen‐independent manner (L. P. Covre, R. G. de Moura, C. H. Fantecelle, P. de Oliveira Lopes, A. Falqueto, A. N. Akbar, & D. C. O. Gomes, unpublished). Therefore, we propose for the first time that senescent CD4‐CLT may be associated with CL immunopathology. The mechanism involved in the tissue damage within the cutaneous lesions of infected individuals comprises cytokine production and direct cytotoxicity.

The initial inflammatory response after parasite infection is important to trigger the leishmanicidal mechanisms,^125^ but it also can induce the expression of stress ligands by the surrounding stroma, including those that bind to NK receptors.^126^, ^127^, ^128^ The induction of stress ligands may lead to nonspecific NK‐related cytotoxic killing and tissue damage that exacerbates inflammation further in a positive feedback loop, promoting a progressive increase in the size of the skin lesions.^129^, ^130^ At this advanced stage, infiltrating senescent CD8^+^ T cells do not have to be specific for *L. braziliensis* but may be able to recognize other antigens (e.g., CMV) that can drive T‐cell senescence and, therefore, the acquisition of NK characteristics. The mechanism that induces the tissue pathology in CL is unclear, however, the wide expression of NK receptors on both senescent CD4^+^ and CD8^+^ T cells in the lesions may be involved and it is essential to determine the presence of different NK ligands for senescent T cells within the tissue that may drive nonspecific pathology. Moreover, identifying the source of inflammation is also pivotal in addressing immuno‐based therapies to improve the patients healing.

### 9.3 The possible role of senescent T cells in different human diseases

The persistent antigenic availability found in autoimmune diseases, organ transplants, and other diseases fosters the continuous activation of antigen‐specific T cells, which leads to the secretion of cytokines and other inflammatory mediators into the tissue environment. The maintenance of the persistent inflammatory environment during nonpathogenic processes has been associated with the accumulation of senescent cells.^131^, ^132^ Furthermore, the proliferative response in the T cell compartment in response to tissues antigens in certain diseases may induce the accumulation of T cells at in late stages of differentiation with senescent characteristics as seen in rheumatoid arthritis,^133^ type 2 diabetes,^134^ multiple sclerosis,^135^, ^136^ and intestinal colitis.^137^ Interestingly, the accumulation of senescent T cells has been associated with the severity observed in patients, suggesting that they may act on the clinical outcome of many diseases such as Amyotrophic lateral sclerosis (ALS), Lupus nephritis, multiple sclerosis, Rheumatoid arthritis Psoriatic arthritis.

There is also evidence that suggests the direct participation of senescent‐like CD4^+^ cytotoxic T cells in promoting pathology. For example, these cells have been linked to the immunopathology dengue hemorrhagic fever (DHF), a severe clinical form of the disease that develops after secondary infection with different dengue viral serotypes from the primary infection.^94^ In this context, senescent‐like CD4^+^‐CTL cross‐reactive to different dengue serotypes are found in patients and demonstrate an increased capacity to mediate the killing of target cells through Fas/FasL recognition or perforin release.^138^, ^139^, ^140^ Identification of two epitopes on the dengue 4 virus capsid protein recognized by a serotype‐specific and a panel of serotype‐cross‐reactive human CD4^+^ cytotoxic T‐lymphocyte clones. Similar findings have been reported in hepatitis virus infection, where senescent‐like CD4^+^ T cells and CD8^+^ T cells expressing perforin that target liver hepatocytes and cause immunopathology are significantly increased in patients compared with healthy controls. Moreover, hepatocytes from HAV‐infected liver overexpressed NKG2D ligands, which was associated with an increased innate‐like cytotoxic function through bystander activation of NKG2D dependent‐manner.

Completely, the hypothetical positive feedback loop described here that involves senescent T cells and excessive inflammation that induces nonspecific tissue damage may also be in operation in other nonresolving inflammatory diseases where these cells accumulate, for example, Chagas disease^141^ and malaria,^142^ cervical cancer,^143^ and Sjogren's syndrome. The accumulation of senescent T cells that have increased pro‐inflammatory potential has also been implicated in age‐related diseases such as rheumatoid arthritis,^144^, ^145^ Alzheimer's,^146^ and cardiovascular diseases.^147^, ^148^ Furthermore, a direct immunopathological role of senescent cells has also been reported in patients with acute hepatitis A infection and this is also associated with the co‐localization of CD8‐NKR‐expressing cells and NKG2D‐ligand‐expressing hepatocytes. However, the NKR‐expressing CD8^+^ T cells required a preactivation step with IL‐15, which was produced by HAV‐infected cells, to induce their killing capacity and liver injury.^149^ Therefore, two signals may be required for tissue damage induced by senescent CD8^+^ T cells, first a preactivation step for the T cells themselves that are induced by cytokines like IL‐15 and second, an inflammatory environment where additional mediators that are present induce NKR‐ligand expression by surrounding tissue.^150^ This mechanism for immunopathology may also occur in other viral infections, such as HIV, COVID, EBV, and respiratory infections, where both the frequencies of terminally differentiated T cells (EMRAs) and systemic inflammation are increased. For example, during SARS‐CoV‐2,^151^, ^152^ Influenza B,^152^ or Seasonal coronavirus,^152^ the frequency of T cells within CD4^+^ and CD8^+^ compartments that express CD57 and KIR or EMRA and inflammation increase significantly in the lungs compared to uninfected individuals.

## 10 HOW CAN SENESCENT STROMAL AND SENESCENT T CELLS BE TARGETED?

The observations that senescent T cells may develop in parallel with senescent tissue cells raises whether the activity of either of the populations can be inhibited to alleviate tissue damage. Preclinical studies of targeting senescent nonlymphoid cells have established that as proof of principle, in animal models, removing senescent cells in tissues cells reduces age‐related deterioration.^152^ In mouse models, senescent cells are identified by expression of the cyclin inhibitor p16Ink4a. Ablation of p16‐expressing cells has been shown to improve metabolic dysfunction in obesity mice and other age‐related disorders, even then applied in late life.^152^, ^153^, ^154^, ^155^ Moreover, clearance of p16‐expressing brain myeloid cells also changed the immune landscape in the brain and restored cognitive function.^156^ This and other evidence indicate the beneficial role of removing senescent cells to reestablish tissue homeostasis. The impact of removing senescent T cells was not investigated in these experiments. Unfortunately, p16 and other cell cycle inhibitor proteins such as p21 that are markedly upregulated in senescent cells of different types are not druggable.

The establishment of pharmacotherapeutic approaches that abolish senescent cells selectively introduced using “senolytic” drugs. The discovery of senolytic drugs has a long history and involved the testing of many strategies to target senescent cells in many different tissues.^158^, ^159^ Successful drug candidates targeted antiapoptotic pathways, specifically in senescent cells. The activity was demonstrated in mice when a short‐term administrate combination of dasatinib and quercetin reduced a significant number of senescent cells and improved survival rate, therefore extending old mice life span.^154^ Long‐term treatment up to 23 months of a combination between dasatinib (D) and quercetin (Q) has shown therapeutic effect in reducing intervertebral disc degeneration by alleviating senescent cell burden in elderly wild‐type C57BL/6 mice.^160^ Interestingly, mice infected with *Leishmania major* and treated with quercetin orally for 28 consecutive days had increased lesional wound‐healing potential compared with control mice. Moreover, they reduced the systemic levels of tumor necrosis factor‐α, interleukin‐6, and adiponectin, as well as superoxide dismutase and glutathione peroxidase activities, suggesting that target senescence cells may be contribute toward reducing *Leishmania* pathology. Once again, the impact of senolytic drugs on senescent T cells was not determined in these experiments, but the results that emerged suggest the participation of the population in the aggravation of many diseases. Another example of using senolytic drugs was in a β‐cell senescent‐induced metabolic disease type 2 diabetes model, using ABT263 (Navitoclax) that reduced SASP release from senescent β‐cell and improved glucose tolerance in mice.^161^ Together, these data signify therapeutic potential for health benefits during aging by using senolytic drugs with well‐tolerated toxicity at least in preclinical models.

A caveat with the use of senolytic drugs is that it is currently unclear what impact they will have on the persistence and function of senescent T cells. If these cells have a beneficial role then removing them may have detrimental consequences (Figure 2A). However, if these cells contribute to immunopathology in certain diseases, then their removal, even temporarily, may allow the destructive inflammatory and tissue damage loop to be broken (Figure 2B). With senolytic therapy in general, it is not clear how long it will take before senescent cells of different types reappear after their initial removal. There are more than 300 clinical trials worldwide for senolytic drugs, especially D + Q, navitoclax, and fisetin (clinicaltrials.gov).^159^ These studies are mostly focusing on age‐related disorders in older subjects aged over 65 years, such as Alzheimer's disease, inflammation‐driven disease, skeletal, and joint disease with the aim to improve quality of life in patients and to reduce frailty.

The first trial in humans was performed in idiopathic pulmonary fibrosis patients, by intermittent dosing of D + Q for 3 weeks.^162^ The result showed improvement in physical and pulmonary function. The SASP measurement for IL6, MMP7, and TIMP2 after treatment exhibits a slightly reduce but is not statistically significant. However, the level was correlated with the patient physical activity. This might be due to short‐term administration and a low number of preexisting senescent cells.^162^ Interestingly, out of numerous clinical trials that were registered in clinicaltrials.gov, only a few studies have mentioned measurements of immune senescent cells (as defined by p16 expression).

Reducing the number of senescent T cells to an optimal level that would give benefit over harm is the most challenging aspect of this therapy. Excessive elimination of the senescent T‐cell burden might disturb tissue homeostasis. The effect of senolytic therapy on circulating senescent T cells is unclear. The drug distributes to blood and any tissues.^162^ Depletion of the cytotoxic population of terminally differentiated cells will possibly interfere with immune responses in older adults, where these populations accumulate.^163^ In addition, interfering with cross talk between senescent stromal and immune cells by senolytic therapy may have an impact on the immune‐mediated clearance of these cells in tissues. Of interest, it was shown that the use of the senolytic drug Quercetin in patients with *Leishmania* reduced the immunopathology in the skin but it was not clear if senescent cells in both lymphoid and nonlymphoid compartments were affected by the treatment.^164^

It was shown recently that inhibiting baseline inflammation in the skin of healthy older humans using an oral p38 MAP kinase inhibitor (Losmapimod from GSK) enhanced the ability of these individuals to respond to an antigen‐specific immune challenge in the skin.^165^ The high level of inflammation was due in part to the secretion of chemokines by senescent fibroblasts, which recruited inflammatory monocytes that then inhibited resident memory T cells in the tissue.^166^ The short‐term p38 inhibition was shown to reduce the SASP secreted by fibroblasts but did not change the number of senescent cells that were present.^166^ Therefore, the impact that senescent stromal cells have on altered immunity in tissues can be reduced by blocking the mediators they secrete without actually removing the cells that are responsible.

Recently, the possibility of using chimeric antigen receptor (CAR)‐T‐cell therapy for targeting senescent cells has raised interest.^168^ CAR‐T is approved to be used in the clinical setting for various immunogenic cancers, for example, multiple myeloma, large B‐cell lymphoma, mantle cell lymphoma, etc. Utilizing its selectivity and efficacy, CAR‐T cells could possibly be exploited to kill senescent cells (or senescent immune cells) more potently with fewer off‐target effects than senolytic drugs. By using urokinase‐type plasminogen activator receptor (uPAR) as an antigen, uPAR CAR‐T cells efficiently kill senescent cells in mice with distinctive condition including chemotherapy‐induced senescence of adenocarcinoma‐bearing mice, and chemically induced liver fibrosis mice.^168^ The data suggest a potential to use CAR‐T in many other age‐related diseases. Identifying new surface antigens that are specific to stromal or immune cells requires further study.^168^

Finally, if senescent T cells in inflamed tissue environments can cause non‐specific immunopathology (Figure 2B) then reducing their NK‐like activity may alleviate this process. In this situation, the inhibition of sestrins in senescent CD8^+^ T cells can switch their function from NK‐like to T‐cell‐like activity.^24^ Sestrin inhibitors may therefore be useful to reduce immunopathology in various diseases. However, inhibiting the sestrins reconstitutes the proliferative activity of senescent T cells, which harbor DNA damage and this may be associated with some risk of malignancy. Nevertheless, the temporary blockade of sestrins may break the positive feedback loop that leads to immunopathology, and this would be of benefit.

## 11 CONCLUDING REMARKS

We live in a world where older individuals are increasing in numbers disproportionately to younger subjects. The increase in life span is not commensurate with an increase in health span. Senescent cells both in tissues and in the immune system accumulate during aging and may have a role in decreased health. Therefore, a better understanding of the biology that underpins their interactive function is essential to determine how the immune system and tissue environments can be targeted individually or together for the benefit of health during aging and in patients with various inflammatory diseases.

## CONFLICT OF INTEREST STATEMENT

The authors have no conflicts of interest to disclose.

## Data Availability

Data sharing not applicable—no new data generated.
